# Health literacy, digital literacy and eHealth literacy in Danish nursing students at entry and graduate level: a cross sectional study

**DOI:** 10.1186/s12912-020-00418-w

**Published:** 2020-04-10

**Authors:** Kamila Adellund Holt, Dorthe Overgaard, Lisbeth Vinberg Engel, Lars Kayser

**Affiliations:** 1 Faculty of Health, Department of Nursing and Nutrition, University College Copenhagen, Copenhagen N, Denmark; 2 Department of Public Health, Section of Health Service Research, University of Copenhagen, Øster Farimagsgade 5, 1353 Copenhagen, Denmark

**Keywords:** Health literacy, Digital literacy, eHealth literacy, Self-rated health, Nursing students, HLQ, eHLQ, eHLA

## Abstract

**Background:**

The increasing number of people living with one or more chronic conditions imposes a growing demand on healthcare providers. One way to handle this challenge is by re-orientating the way care is provided, empower people and increase their ability to manage their condition. This requires, amongst other factors, sufficient level of health literacy (HL) and digital competences among both patients and the healthcare providers, who serve them. The focus of this study is the level of HL, digital literacy (DL), and eHealth literacy (eHL) in nursing students in Denmark.

The objective was to examine the level of these three literacies in entry- and graduate-level nursing students and examine sociodemographic characteristics and self-rated health (SRH) associations.

**Methods:**

A cross sectional study was conducted among 227 students at entry-level and 139 students at graduate-level from a nursing program. The survey consisted of the health literacy questionnaire (HLQ (nine scales)), the eHealth Literacy Assessment toolkit (eHLA (seven scales)), the eHealth Literacy Questionnaire (eHLQ (seven scales)), questions soliciting sociodemographic data, and a single item assessing the students’ SRH. Pearson’s chi-square test and the Mann-Whitney test were used to examine the differences in HL, DL, and eHL and between groups, and Kendall’s tau-b test to examine correlations between SRH and HL, DL, and eHL.

**Results:**

The level of HL, DL and eHL tended to be higher among graduate-level students than in entry-level students and was satisfactory. Age, sex, country of origin, and parents’ educational level and occupational background influenced students’ HL levels. SRH was higher in students at the graduate level. Amongst entry-level students, SRH was positively associated to seven HLQ, four EHLA and four eHLQ, amongst graduate-level students, SRH was positively associated to seven HLQ and six eHLQ.

**Conclusions:**

Educators must be aware of how sociodemographic factors affects students’ literacies and increase learning opportunities by mixing students when planning activities. Considering the higher SRH in graduate-level students, HL, DL, and eHL levels indicate that current curricula and study activities are appropriate, but there is still room for improvement.

## Background

The increasing number of elderly people and the growing prevalence of lifestyle-associated non-communicable diseases calls for new actions to engage and empower people to take better care of their health [1]. The ultimate goal is to support patient’s well-being and self-management by providing education and involving patients in joint (shared)-decision making [2, 3]. This requires a sufficient level of health literacy (HL) for both patients and those who serve people living with chronic conditions [4].

The focus of health service providers must be on how to increase HL and how the workforce can foster a more supportive environment where patients can navigate easily.

This requires healthcare workers to be aware of both the concept of HL and how the digitalisation can either impose a barrier or be a facilitator in the provision of care. To understand the digital aspect the health care workers also need to have an understanding of the patient’s digital literacy (DL) and eHealth Literacy (eHL) [5, 6].

Education plays a significant role in the understanding of health literacy among healthcare workers. In particular, nurses need new competencies, including HL, DL, and eHL, as they take on new roles and responsibilities related to digital health transformation and re-orientation of healthcare [7] and help patients navigate between allied health professionals. Consequently, over the past decade, universities and colleges worldwide have increasingly had a focus on awareness among nurses of the importance of patients’ HL levels [8, 9], as well as aspects of nursing students’ HL levels [9–11] and digital competences [12–14], addressing these aspects as part of the curriculum [7, 15].

Currently, little is known about HL, DL and eHL levels among nursing students and how they are influenced by academic levels and sociodemographic characteristics. Studies using the Health Literacy Questionnaire (HLQ) [11] and the Adult Health Literacy Scale (AHLS) [10] have demonstrated higher HL among nursing students at the graduate level, compared to the entry level. Zou et al. found higher scores in three HLQ domains in undergraduate students aged 20–24 years but did not examine students at the graduate level [11]. The sex of nursing students is unrelated to their HL level [10, 11]. AHLS scores were higher among nursing students with a chronic condition or taking medication [10]. Zou et al. also found that undergraduate nursing students with chronic conditions reported were better at finding good health information and better to understand health information well enough to know what to do, but they were not better than those without a chronic condition at appraising health information [11]. HL may positively be associated with parental education level and socio-economic status [11]. Data on the relationship between students’ geographical background and HL are scarce; however, urban vs. rural residency did not influence HL [10, 11].

Findings are conflicting in regard to the influence of academic level on eHL. Two studies using the eHealth Literacy Scale (eHEALS) [16] reported a correlation between eHL and academic level [17, 18] that did not appear to be related to age [18], whereas a recent study from Sri Lanka found no association between academic level or age and eHL [19]. The sex of nursing students was unrelated to eHL levels [18, 19].

The introduction of multi-facetted instruments to measure HL, DL, and eHL creates new opportunities to obtain better insight into nursing students’ competences. In this study, we used the HLQ [20], eHealth Literacy Assessment (eHLA) toolkit [21], and eHealth Literacy Questionnaire (eHLQ) [22] to measure HL, DL and eHL respectively. The HLQ was selected as it recently has been used in a global initiative to measure HL in various regions of the world, primarily among nursing students [23]. To the best of our knowledge, neither the eHLA nor the eHLQ have been previously used among students, nursing or otherwise. Both were recently used to investigate eHL in a medical outpatient clinic [24]. This approach supports gaining insight into nursing students’ self-reported capability to navigate and act in the healthcare sector.

In 2013, the World Health Organization (WHO) reported a positive correlation between self-rated health (SRH) and HL [25]. SRH is a reliable indicator of health status and a strong predictor of mortality over time [26]. Little is known about SRH in nursing students in relation to HL. Hsu et al. found that medical students, who had better perceived health and paid more attention to their health, were more likely to seek and evaluate health information and had a higher level of eHL [27]. Students in nursing programs receive a thorough education in health that might increase their HL and ability to manage their own health [27] resulting in a high level of SRH. On the other hand, pressure from school and clinical work may lead to stress, anxiety, and reduced SRH [28].

The aim of this study was to answer the following research questions:

1. What are the levels of HL, DL, and eHL among students entering a nursing program?
2. What are the levels of HL, DL, and eHL in entry-level versus graduate-level nursing students?
3. Is there an association between the literacies and the sociodemographic characteristics or health conditions?
4. Is SRH different in graduate-level students compared to students at entry-level?
5. Are there any associations between SRH, and HL, DL and eHL respectively in entry- or graduate-level students?

## Methods

### Study design and participants

**Fig. 1:**
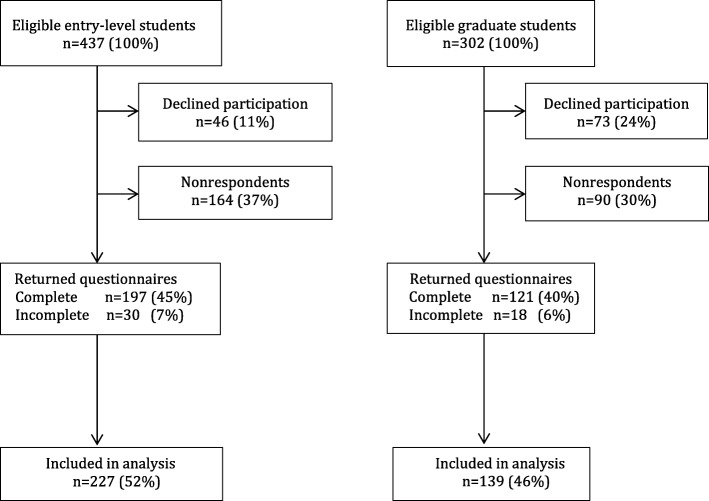
Participant flow

A cross sectional study was conducted in February through May 2017 among entry- and graduate-level students in the nursing program at University College Copenhagen, Denmark. After an oral presentation and written information provided by email, all enrolled students at 1st (entry-level) and 7th semester (graduate-level) of the nursing program were invited to participate via an e-mail containing a link to the survey (provided as supplementary file). The students were informed that they provided consent to participate in the study by completing the survey, which was hosted online by Enalyzer Software A/S (Copenhagen, Denmark). Students who did not respond received up to four reminders by email. An incentive to complete the survey was provided by offering a free cup of coffee to the first 160 entry-level students who responded. The first six graduate-level students who responded were offered a cinema ticket. Figure 1 depicts the participant flow where the response rate was 50% (366/739).

### Survey instrument

The survey consisted of the HLQ, the eHLA toolkit, the eHLQ, questions soliciting sociodemographic data, and a single item assessing students’ SRH.

### HLQ

The HLQ is developed based on a conceptual model [20] and has been widely used in many languages including studies in nursing and other college students [23]. We used a validated and cultural adapted Danish version of the HLQ [29]. The HLQ consists of 44 items addressing 9 conceptually distinct domains of HL: 1) feeling understood and supported by healthcare providers, 2) having sufficient information to manage my health, 3) actively managing my health, 4) social support for health, 5) appraisal of health information, 6) ability to actively engage with healthcare providers, 7) navigating the healthcare system, 8) ability to find good health information, and 9) understanding health information well enough to know what to do. Response options for subscales 1–5 range from 1 (strongly disagree) to 4 (strongly agree) and options for subscales 6–9 range from 1 (very difficult) to 5 (very easy).

### The eHLA toolkit

The eHLA toolkit was developed in the period of 2011 to 2015 where the scales were continuously tested and developed in an iterative process to ensure content and face validity [21]. The toolkit consists of 44 items grouped into 7 tools. Tools 1–4 assess HL and tools 5–7 assess DL. The tools 1, 2, 5 and 6 build on established questionnaires. Tool 1, 5 and 6 have been redesigned, where tool 2 builds on items from the HLS-EU instrument [30]. Tools 3,4 and 7 have been developed from scratch. All tools have been thoroughly explored and validated using modern test theory [21]. The instrument is developed in Danish. Tools and response options are as follows:

1. Functional health literacy, 10 items scored as the sum of correct answers (1 to 10)
2. Self-assessed health literacy, nine items, four response options from very difficult to very easy calculated as mean range (1 to 4)
3. Familiarity with health and health care, five items, response options from 1 (no knowledge) to 4 (complete knowledge)
4. Knowledge of health care, six items, correct answers receive two points, incorrect answers receive zero points, and opting out receives one point, item scores are summed (1 to 12)
5. Familiarity with technology, six items, response options from 1 (not at all familiar) to 4 (completely familiar)
6. Technology confidence, four items, response options from 1 **(**completely uncertain) to 4 (absolutely sure)
7. Incentives for engaging with technology, four items, response options from 1 (strongly disagree) to 4 (strongly agree)

### eHLQ

The eHLQ is developed based on the eHealth literacy framework [31], which is a conceptualization of factors important to consider when people use digital technology and services in relation to their health. The eHLQ is developed concomitantly in Danish and English using both classical and modern test theory [22]. The instrument is currently licensed for usage in more than 30 studies in more than 12 countries. The on-going translations and cultural adaptations indicate that the instrument is robust across various contexts.

The eHLQ consists of 35 items in seven domains [22]: 1) using technology to process health information, 2) understanding of health concepts and language, 3) ability to actively engage with digital services, 4) feel safe and in control, 5) motivated to engage with digital services, 6) access to digital services that work, and 7) digital services that suit individual needs. Domains 1–5 consist of 5 items, domain 6 consists of 6 items, and domain 7 consists of 4 items. Response options for all items range from 1 (strongly disagree) to 4 (strongly agree).

### Sociodemographic data

Items soliciting sociodemographic data included age measured as a continuous variable and seven dichotomous variables: sex (male/female), country of birth (Denmark or other), whether Danish was spoken at home (yes/no), whether the respondents’ parents worked in social or healthcare fields (yes/no), previous experience with being hospitalized or receiving treatment in an outpatient clinic (yes/no), chronic conditions (yes/no), and use of daily prescription medications including birth control pills (yes/no). The educational levels of students and their parents were measured as separate categorical variables with six response options: public school, general upper secondary education, vocational education training, short-cycle higher education (less than 3 years), medium-cycle higher education (3–4 years), and long-cycle higher education (more than 5 years) [32].

### SRH

SRH was rated on a 5-point Likert scale from excellent to poor [33, 34].

### Descriptive statistics

Data from the HLQ, eHLQ scales, eHLA tools and SRH were reported as means and interquartile ranges. Scores on HLQ and eHLQ scales and eHLA tools 2, 3, and 5–7 are calculated as the mean of item scores in the domain or tool. If less than 50% of items were completed, the value for the domain or tool was not calculated. If at least 50% of items were completed, a mean score for the domain or tool was calculated by replacing scores for missing items with the mean score for completed items. The scale for SRH was reversed for the statistical analysis, thus reporting of excellent condition was scored as 5 and poor as 1.

### Tests statistics

Pearson’s chi-square test was used to test for differences between entry- and graduate-level students with respect to the following variables: sex, born in Denmark, speak Danish at home, parents working within social and health care, being a patient at the hospital, suffering from a chronic condition and taking prescribed medication. The non-parametric Mann-Whitney test was used to test for differences between entry- and graduate-level students with respect to the level of education, parents’ education, SRH, HL, DL, and eHL. The Mann-Whitney test was also used to test the differences in the level of HL, DL, and eHL respectively within the following categories: country of birth, use of the Danish language used at home, previous hospitalization or outpatient treatment, use of daily prescribed medication, chronic condition, and parental employment in the social or healthcare system.

Associations between HL, DL, and eHL and age, SRH, and student and parental educational level were examined using Kendall’s tau-b non-parametric test. We interpreted the strength of the correlation according to Brace (weak ≤ ± 0.2, moderate ±0.3 to 0.6, strong ≥ ± 0.7) [35]. We used IBM Corp. released 2013, IBM SPSS Statistics for Windows, Version 22.0. Armonk, NY.

## Results

### Participant characteristics

Mean age was 24.6 (IQR 21–25) years among entry-level students and 26.8 (IQR 24–28) years among graduate-level students. As seen in Table 1, most students were young females. More than half of the parents had a medium or long-cycle higher education. Significantly more entry-level students (164, 58.8%) than graduate-level students (115, 41.2%) had been a patient in a hospital or received outpatient treatment (*Χ*^2^ = 6.529, *p* = .011). Overall, entry-level students had a lower educational level than graduate-level students (*Χ*^2^ = 19.923, *p* = .001), of whom several had medium-term higher education.

**Table 1:** Participant characteristics, number (percentage)

	*N* = 366
Sex	
Male	29 (7.9%)
Female	337 (92.0%)
Born in Denmark	330 (90.1%)
Speak Danish as primary language at home	345 (94.3%)
One of parents work or has worked within social or healthcare	167 (45.6%)
Students’ highest educational level	
Public school	1 (0.3%)
General upper secondary education	259 (70.8%)
Vocational training	11 (3.0%)
Short-cycle higher education (below 3 y.)	40 (10.9%)
Medium-cycle higher education (3–4 y.)	50 (13.7%)
Long-cycle higher education (above 5 y.)	3 (0.8%)
Parents’ highest educational level	
Public school	15 (4.1%)
General upper secondary education	18 (4.9%)
Vocational training	94 (25.7%)
Short-cycle higher education (below 3 y.)	41 (11.2%)
Medium-cycle higher education (3–4 y.)	120 (32.8%)
Long-cycle higher education (above 5 y.)	76 (20.8%)
Previous hospitalization or outpatient clinic treatment	279 (76.2%)
Chronic condition	77 (21.0%)
Daily use of prescribed medication	209 (57.1%)

Among both entry- and graduate-level students, 77 (21%) reported that they suffered from a chronic condition and 209 (57.1%) took prescribed medication daily.

HL level was higher among graduate-level students than among entry-level students in all domains except HLQ1, feeling understood and supported by healthcare providers (Table 2)*.*

**Table 2:** HLQ levels among entry- and graduate-level nursing students

HLQ – scale	Entry-level students	n	Graduate-level students	n	*P* value^1^
	Mean (Q_1_-Q_3_)		Mean (Q_1_-Q_3_)		
1. Feeling understood and supported by healthcare providers	2.96 (2.75–3.25)	206	2.93 (2.50–5.50)	123	.604
2. Having sufficient information to manage my health	3.07 (3.00–3.25)	206	3.29 (3.00–3.75)	123	**.000**
3. Actively managing my health	2.80 (2.40–3.00)	204	2.95 (2.60–3.20)	122	**.003**
4. Social support for health	3.29 (3.00–3.80)	206	3.33 (3.00–3.80)	123	.388
5. Appraisal of health information	2.83 (2.60–3.00)	204	3.02 (2.80–3.25)	122	**.000**
6. Ability to actively engage with healthcare providers	3.80 (3.40–4.20)	202	3.87 (3.60–4.20)	121	.241
7. Navigating the healthcare system	3.70 (3.50–4.00)	202	3.84 (3.58–4.16)	121	**.012**
8. Ability to find good health information	4.07 (3.80–4.20)	202	4.25 (4.00–4.60)	121	**.000**
9. Understand health information well enough to know what to do	3.97 (3.80–4.20)	199	4.18 (4.00–4.40)	121	**.000**

Statistically significant results are **bolded**.

Graduate-level students scored higher than entry-level students on 5 of the 7 eHLA tools. There were no between-groups differences for eHLA2, health literacy self-assessment and the eHLA6 digital literacy tool*,* technology confidence (Table 3).

**Table 3:** eHLA levels among entry- and graduate-level nursing students

eHLA – tool	Entry-level students	n	Graduate-level students		
	Mean (Q_1_-Q_3_)		Mean (Q_1_-Q_3_)	n	*P* value^1^
1. Functional health literacy	9.09 (8.00–10.00)	198	9.66 (9.00–10.00)	121	**.000**
2. Health literacy performance	3.10 (2.88–3.00)	197	3.18 (2.88–3.55)	121	.180
3. Health literacy knowledge	2.28 (1.80–2.60)	197	2.64 (2.20–3.00)	121	**.000**
4. Health literacy self-assessment	9.81 (9.00–11.00)	197	11.63 (12.00–12.00)	121	**.000**
5. Computer incentives	3.46 (3.16–4.00)	197	3.61 (3.33–4.00)	121	**.013**
6. Familiarity	3.52 (3.25–4.00)	197	3.64 (3.50–4.00)	121	.080
7. Computer confidence.	3.41 (3.00–4.00)	197	3.54 (3.25–4.00)	121	**.017**

Statistically significant results are **bolded**.

Graduate-level students scored higher than entry-level students on eHLQ1–3, which pertained to personal knowledge and skills. There were no differences between graduate- and entry-level students on the other eHLQ domains, which pertained to the interface and experience with healthcare services (Table 4).

**Table 4:** eHLQ levels among between entry- and graduate-level nursing students

eHLQ – dimension	Entry-level students	n	Graduate-level students	n	*P* value^1^
	Mean (Q_1_-Q_3_)		Mean (Q_1_-Q_3_)		
1. Using technology to process health information	2.81 (2.60–3.00)	213	2.94 (2.60–3.20)	127	**.010**
2. Understanding of health concepts and language	3.08 (3.00–3.20)	213	3.37 (3.00–3.80)	127	**.000**
3. Ability to actively engage with digital services.	2.98 (2.80–3.20)	222	3.23 (3.00–3.60)	131	**.000**
4. Feel safe and in control	3.03 (2.80–3.20)	213	3.07 (2.80–3.20)	127	.318
5**.** Motivated to engage with digital services	2.76 (2.40–3.00)	213	2.81 (2.60–3.00)	127	.305
6. Access to digital services that work	2.81 (2.66–3.00)	213	2.85 (2.50–3.16)	127	.494
7. Digital services that suit individual needs	2.73 (2.50–3.00)	208	2.81 (2.50–3.00)	127	.222

Statistically significant results are **bolded**

### Association between sociodemographics and literacy among entry-level nursing students

Age was associated with 5 of 23 investigated literacy domains. Two HL domains were positively but weakly correlated with age: HLQ3*,* actively managing my health (tau-b. = .155, *p* = .003) and eHLA4, knowledge of health and disease (tau-b = .202, *p* = .000). Three domains related to DL or eHL were negatively but weakly correlated with age: eHLQ4, feel safe and in control (tau-b = −.107, *p* = .038); eHLA5, technology familiarity (tau-b = −.145, *p* = .006); and eHLA6, technology confidence (tau-b = −.117, *p* = .032).

Sex was associated with literacy on 2 of 23 literacy domains. The mean score was higher for males than for females on HLQ 6, ability to actively engage with healthcare providers (males 4.16, IQR: 3.80–4.60 vs. females 3.77, IQR: 3.40–4.00, z = − 2.47, *p* = .014) and eHLA5, technology familiarity (males 3.77, IQR: 3.66–4.00 vs. females 3.43, IQR: 3.00–3.83, z = − 2.79, *p* = .005).

Country of birth was associated with literacy on 4 of 23 domains. Participants, who were born in Denmark, scored higher than those who were born elsewhere on 3 items: HLQ4, social support for health (mean 3.32, IQR: 3.00–3.80 vs. 3.02, IQR: 2.65–3.55, z = − 1.97, *p* = .048); eHLA1, functional health literacy (mean 9.14, IQR: 9.00–10.00 vs. 8.65, IQR: 8.00–9.00, z = − 2.33, *p* = .020) and eHLA5, technology familiarity (mean 3.49, IQR: 3.16–4.00 vs. 3.20, IQR: 2.83–3.83, z = − 2.09, *p* = .037). Participants who were born in Denmark scored lower than those who were born elsewhere on eHLA3, familiarity with health and health care (mean 2.24, IQR: 1.80–2.60 vs. 2.63, IQR: 2.00–3.20, z = − 2.32, *p* = .020). No between-group differences in any domains existed for Danish as primary language at home.

Participants who had at least one parent with work experience in the social or healthcare system scored higher than those whose parents had not worked in the social or healthcare sectors on HLQ1*,* feeling understood and supported by healthcare providers (mean 3.10, IQR: 2.75–3.50 vs. 2.83, IQR: 2.50–3.00, z = − 3.22, *p* = .001) and eHLA1, functional health literacy (mean 9.20, IQR: 9.00–10.00 vs. 8.98, IQR: 8.00–10.00, z = − 2.11, *p* = .035).

Students’ educational levels before nursing program entry were associated with literacy on 3 of 23 domains. Positive but weak correlations were found for HLQ3, actively managing my health (tau-b = .166, *p* = .005); eHLA3, familiarity with health and health care (tau-b = .133, *p* = .024); and eHLA4, knowledge of health and disease (tau-b = .195, *p* = .001). Students’ educational level was negatively but weakly correlated with HLQ4, social support for health (tau-b = −.121, *p* = .039). Parental educational level was not correlated with any literacy domains.

Entry-level nursing students who had been hospitalized or received treatment in an outpatient clinic scored higher than those who had not on eHLQ4*,* feel safe and in control *(*mean 3.06, IQR: 2.80–3.20 vs. 2.93, IQR: 2.80–3.00, z = − 2.11, *p* = .035*)* and eHLQ*6,* access to digital services that work *(*mean 2.85, IQR: 2.66–3.00 vs. 2.72, IQR: 2.50–3.00*,* z = − 2.85, *p =* .004*).* Students with a chronic condition had lower scores than those who did not on HLQ6, ability to actively engage with healthcare providers *(*mean 3.63, IQR: 3.15–4.00 vs. 3.85, IQR: 3.60–4.20*,* z = − 2.46, *p* = .014*);* eHLA5, technology familiarity *(*mean 3.33, IQR: 3.00–3.83 vs. 3.49, IQR: 3.16–4.00, z = − 1.98, *p* = .048*);* and eHLA6, technology confidence *(*mean 3.34, IQR: 3.00–7.75 vs. 3.56, IQR: 3.25–4.00*,* z = − 2.89, *p* = .004*).*

Similarly, students who used prescribed medication on a daily basis scored lower than those who did not on HLQ8*,* ability to find good health information (mean 4.02, IQR: 3.80–4.20 vs. 4.14, IQR: 4.00–4.40, z = − 2.11, *p* = .035); HLQ9*,* understand health information well enough to know what to do (mean 3.91, IQR: 3.60–4.05 vs. no 4.06, IQR: 3.80–4.20, z = − 2.41, *p* = .016); and eHLA2*,* health literacy self-assessment (mean 3.04, IQR: 2.88–3.22 vs. 3.18, IQR: 3.00–3.44, z = − 2.39, *p* = .017).

### Association between sociodemographics and literacy among graduate-level nursing students

Age was not associated with literacy among graduate-level students. Males scored higher than females on HLQ1*,* feeling understood and supported by healthcare providers (mean 3.46, IQR: 3.00–4.00 vs. 2.90, IQR: 2.50–3.25, z = − 2.08, *p* = .038); eHLA5*,* technology familiarity (mean 3.88, IQR: 4.00–4.00 vs. 3.59, IQR: 3.33–4.00, z = − 2.11, *p* = .035); and eHLA6*,* technology confidence (mean 3.85, IQR: 4.00–4.00 vs. 3.63, IQR: 3.50–4.00, z = − 2.06, *p* = .040).

Students who were not born in Denmark scored lower than those who were born in Denmark on 4 of 13 HL domains: HLQ2*,* having sufficient information to manage my health (mean 3.32, IQR: 3.00–3.75 vs. 2.92, IQR: 2.68–3.06, z = − 3.05, *p* = .002); HLQ4*,* social support for health (mean 3.37, IQR: 3.00–3.80 vs. 2.80, IQR: 2.55–3.05, z = − 3.45, *p* = .001); HLQ9*,* understand health information well enough to know what to do (mean 4.20, IQR: 4.00–4.40 vs. 3.92, IQR: 3.55–4.25, z = − 2.06, *p* = .039); and eHLA2*,* health literacy self-assessment (mean 3.21, IQR: 2.88–3.55 vs. 2.94, IQR: 2.66–3.00, z = − 2.31, *p* = .021). Students who did not speak Danish as primary language at home scored significantly lower than those who did on HLQ2*,* having sufficient information to manage my health (mean 3.31, IQR: 3.00–3.75 vs. 2.91, IQR: 2.68–3.06, z = − 2.33, *p* = .020); HLQ3*,* actively managing my health (mean 2.97, IQR: 2.65–3.20 vs. 2.46, IQR: 2.15–3.00, z = − 2.26, *p* = .024); and HLQ4*,* social support for health (mean 3.36, IQR: 3.00–3.80 vs. 2.73, IQR: 2.55–3.00, z = − 2.98, *p* = .003).

Students’ educational levels before entering the nursing program were positively but weakly correlated only with eHLA3, familiarity with health and health care (tau-b = .187, *p* = .013). Parental educational levels were positively, moderate correlated with eHLQ*2,* understanding of health concepts and language (tau-b = .211, *p* = .003) and weakly correlated to eHLQ3*,* ability to actively engage with digital services (tau-b = .139, *p* = .043). Parental work in the social or healthcare system was not associated with measured literacy domains.

Students who had been hospitalized or visited an outpatient clinic scored lower than those who had not on eHLQ7*,* digital services that suit individual needs (mean 2.76, IQR: 2.50–3.00 vs. 3.02, IQR: 2.75–3.25, z = − 2.19, *p* = .028). Having a chronic condition and using prescribed medication daily were not associated with measured literacy domains.

### SRH in entry- and graduate-level students

Graduate-level nursing students had a higher SRH level than entry-level nursing students (mean 3.95, IQR: 3.50–5.00 vs. 3.84, IQR: 3.00–4.00, *p* = .001).

### SRH and HLQ

Among entry-level students, SRH was positively correlated with 7 of 9 HLQ domains. Among graduate-level students, SRH was positively correlated with 5 HLQ domains. For entry- and graduate-level students alike, HLQ1, feeling understood and supported by healthcare providers and HLQ8, ability to find good health information, were not related to SRH. For graduate-level students, HLQ2, having sufficient information to manage my health and HLQ9, understand health information well enough to know what to do, were not associated with SRH (Table 5).

**Table 5:** Correlation between SRH and HLQ domains among all participants

	N	Entry-level students	N	Graduate-level students
1. Feeling understood and supported by healthcare providers	206	0.013	123	−0.001
2. Having sufficient information to manage my health	206	**0.228**	123	0.085
3. Actively managing my health	204	**0.23**	122	**0.224**
4. Social support for health	206	**0.202**	123	**0.257**
5. Appraisal of health information	204	**0.199**	122	**0.153**
6. Ability to actively engage with healthcare providers	202	**0.19**	121	**0.189**
7. Navigating the healthcare system	202	**0.151**	121	**0.178**
8. Ability to find good health information	202	0.093	121	0.092
9. Understanding health information well enough to know what to do	199	**0.157**	121	0.03

Note: Correlation assessed with Kendall’s tau-b nonparametric test

Statistically significant results are **bolded**. The strength of the correlation (weak ≤ ± 0.2, moderate ±0.3 to 0.6, strong ≥ ± 0.7)

### SRH and eHLA

Among entry-level students, SRH was correlated with four domains: eHLA2, health literacy self-assessment; eHLA5, technology familiarity; eHLA6, technology confidence; and eHLA7, incentives for engaging with technology (Table 6). No relationship was found between SRH and eHLA among graduate-level students*.*

**Table 6:** Correlation between SRH and eHLA domains among all participants

	N	Entry-level students	N	Graduate-level students
1. Functional health literacy	198	0.026	121	−0.063
2. Health literacy self-assessment	197	**0.12**	121	0.105
3. Familiarity with health and healthcare	197	−0.02	121	−0.046
4. Knowledge of health and disease	197	−0.056	121	0.058
5. Technology familiarity	197	**0.196**	121	−0.007
6. Technology confidence	197	**0.182**	121	−0.025
7. Incentives for engaging with technology	197	**0.17**	121	0.098

Note: Correlation assessed with Kendall’s tau-b nonparametric test

Statistically significant results are **bolded**

### SRH and eHLQ

Among entry-level students, SRH was positively correlated with 4 of 7 eHLQ domains (Table 7). Among graduate-level students, it was positively correlated with 6 eHLQ domains. For both entry- and graduate-level students, eHLQ2, understanding of health concepts and language was not related to SRH. In addition, for entry-level students, eHLQ1*,* using technology to process health information and eHLQ4, feel safe and in control were not correlated with SRH.

**Table 7:** Correlation between SRH and eHLQ domains among all participants

	N	Entry-level students	N	Graduate-level students
1. Using technology to process health information	213	0.073	127	**0.145**
2. Understanding of health concepts and language	213	0.044	127	0.109
3. Ability to actively engage with digital services	222	**0.149**	131	**0.209**
4. Feel safe and in control	213	0.089	127	**0.205**
5. Motivated to engage with digital services	213	**0.136**	127	**0.164**
6. Access to digital services that work	213	**0.132**	127	**0.222**
7. Digital services that suit individual needs	208	**0.16**	127	**0.307**

Note: Correlation assessed with Kendall’s tau-b nonparametric test

Statistically significant results are **bolded**

## Discussion

### Principal findings

This is the first study to report on entry- and graduate-level nursing students’ HL, DL, and eHL and to explore associations with sociodemographic factors and SRH.

HL is higher among graduate-level students compared to entry-level students except in the domains of feeling understood and supported by healthcare providers and self-assessed health literacy. This may indicate that, even though students improve knowledge and information handling over time, they grow uncertain. This positive association between HL and academic level is also documented in other studies using HLQ or AHLS [10, 11].

The perception of and experience with digital services (eHLQ4-eHLQ7) were not higher among graduate-level students. This may be due to their clinical experiences in using electronic systems which have not yet assured them of the benefit of digital services and technology in clinical settings.

Higher scores in three eHLQ scales relating to personal knowledge and skills (eHLQ1-eHLQ3) among graduate-level students align with previous studies that found a higher eHEALS score among nursing students, compared to pre-nursing students [17, 18]. It is noteworthy that a 2019 study from Sri Lanka did not find a positive association between academic level and eHL using eHEALS [19]. This may be explained by differences in curricula and digital maturity between the settings.

### Sociodemographic findings

From a nurse educators’ perspective being aware of possible implications of students’ background is essential. We found that sex, ethnicity, parental education level, socio-economic status and health condition influence students’ HL, DL or eHL.

### Age

Information about the influence of age on health literacy among nursing students is sparse [11]. We evaluated the association of age with HL at both entry and graduate levels and found only two domains associated with age among entry-level students. The inverse relationship between age and two DL tools among entry-level, but not graduate-level students may be due to younger students feeling more familiar and confident with computers than older students at the entry level; a difference that disappears at the graduate level. The inverse relationship between age and feeling safe and in control among entry-level students may be due to increased skepticism among older entry students.

Our findings are in contrast to reports by Tubaishat and Habiballah [18] and Rathnayake [19], who found no relationship between age and eHEALS. This may be due to the use of different instruments.

### Sex

Previous reports on HL in nursing students using HLQ only focused on associations between sex and HLQ domains 5, 8, and 9, thus our findings are not directly comparable. On the other hand, our finding, that sex does not affect HL, is consistent with previous studies [10, 11]. Males tended to score higher in the DL domains of familiarity and confidence, which is in contrast to previous reports that found no sex differences in eHEALS scores [18, 19]. However, our results should be interpreted with care because less than 10% of participants were male.

### Ethnicity

Challenges for students not born in Denmark may occur. We found that this subgroup of entry-level students had lower functional health literacy, felt less supported in relation to health, and reported less familiarity with computers.

At the graduate level, this group was challenged by not having sufficient information to manage their health and not feeling that they understood health information well enough to know what to do. Similar to entry-level students, graduate-level students not born in Denmark may find less support for health among their family and friends, which may contribute to lower self-reported HL scores.

Students who did not speak Danish at home had an additional challenge. They reported lower scores in three HLQ domains (HLQ2-HLQ4) at the graduate level, which indicates a sense of not having enough information and not being able to actively manage their own health and feeling a lack of social support by family and friends. It should be noted that this difference only exists for graduate-level students. The years spent in nursing school may have imposed doubt about their own competences. Underlying reasons regardless, it is important for the nurse educator to be aware of potential pressure caused by low HL among graduate students, who do not speak Danish at home. Further studies are needed to explore how the country of birth and language spoken at home may influence nursing students and students in other health education programs in relation to their confidence and perceived competence.

### Parents’ health-related work and educational level

Parents’ work within healthcare did not affect graduate-level students’ literacy levels. However, it was associated with higher functional health literacy and feeling better understood and supported by health professionals among entry-level students. Entry-level students may perceive health professionals as being like their parents or have more exposure to health knowledge and information at home. Parents’ educational level correlated only with the domains of understanding health concepts and language and ability to actively engage with digital services but did not correlate with any other literacy domains. The finding that HL is unrelated to parental educational background is in contrast to Zou et al. [11], who found that HLQ5, HLQ8 and HLQ9 are associated with parents’ educational and socioeconomic status.

### Previous hospitalization, chronic condition and medication

We found no association between HL and students’ experiences with hospitalization or outpatient treatment but higher scores for the eHLQ domains of feeling safe and in control (eHLQ4), and access to digital services that work (eHLQ6). However, graduate-level students had lower scores for digital services that suit individual needs (eHLQ7). These findings may be explained by positive experiences with the various digital services they encounter as patients whereas the nursing students without this clinical insight may be influenced by negative reporting from the media. The lower score among graduate-level students may reflect a more critical attitude after direct contact with digital services as a health professional.

Our finding that only 1 of 13 HL domains differed between those entry-level students with and without a chronic condition is in contrast to previous reports by Ayaz-Alkaya and Terzi, who found that students with a chronic condition scored higher in AHLS, and Zou et al., who found higher HLQ8 and HLQ9 scores among students with a chronic condition [10, 11]. It is surprising that students with a chronic condition had lower scores on two of the three DL scales as the questions asked are not health related. We would have expected that the students with a chronic condition would be more confident and familiar with technology as their condition might be an incentive to use apps and smartphones in relation to their own health. In contrast to those in contact with hospitals, the entry-level students with a chronic condition had lower scores in three of the domains (HLQ6, eHLA5, eHLA6). This implicates that students who live with a chronic condition not requiring hospital contact may perceive the digital and health care services differently.

The only association between taking prescribed medication and the literacies were found in entry-level students, who had lower scores in HLQ8, HLQ9 and eHLA2. We have no apparent explanation for these findings at entry level, where we would have expected that students having regular contact to health professionals should score higher. The relatively high number of students taking prescribed medication may be explained by the inclusion of birth control pills in the question. In this way the answer may not reflect that the students need the medication for a specific health condition.

### Self-rated health

Graduate-level students had higher SRH than entry-level students, which may be the result of the general increase in HL, DL and eHL. Whether or not there are any causal relationships between students’ literacy and SRH is not evident. Our analysis of associations between domains and SRH in both entry- and graduate-level students do not provide us with a clear pattern. HLQ1, HLQ8, eHLA1, eHLA3, eHLA4, and eHLQ2 are not related to SRH in entry- or graduate-level students indicating that these domains are not related to how students perceive their health. HLQ3–7, eHLQ3, and eHLQ5–7 are related to SRH in both groups. This demonstrates a consistent association which is not related to the nursing program, but already exists at entry-level.

The lack of correlation between SRH in students with the DL tools (eHLA5–7) is surprising because the SRH is correlated with six of the seven eHLQ scales. Associations between DL, eHL and SRH in entry-level students are similar to those in a recent study by our group in an outpatient clinic, where we found the same pattern, with eHLA2 and eHLA5–7 associated with SRH [24]. In addition, eHLQ3, eHLQ5, and eHLQ6 correlated positively with SRH [24].

SRH data for graduate-level students should be interpreted with great care because 22.1% of these respondents marked the response “excellent”, which may lead to a pronounced ceiling effect.

Although most graduate-level students, who has been in clinical training, have satisfactory levels of SRH, HL, DL, and eHL, they should be aware of the challenges they will face in clinical practice when they graduate, and how these can negatively influence their SRH [28]. Future studies are needed to explore whether graduated nurses today are less prone to experience decreased SRH when facing the clinical responsibilities and tasks than the nurses who completed their training 10 years ago. Importantly, new nurses may be less likely to involve colleagues if they need support, because the only HLQ domain that was not scored higher by graduate, compared to entry-level, students was HLQ1*,* feeling understood and supported by healthcare providers.

### Limitations of the study

We only examined nursing students and therefore limited our use of literature for the background and analysis to studies examining only nurses, excluding those that did not clearly include nursing students [36–38]. We also excluded literature on information literacy. This narrowed our scope, but also eliminated many confounders that would have been introduced, if we tried to understand our findings in relation to other professions.

Another limitation is the cross sectional design, in which we studied both entry- and graduate-level students at the same time. The two populations are not fully comparable with respect to sociodemographic characteristics. Observing changes in the same students over time in a longitudinal study would be a stronger design. As noted above, the ceiling effect for the SRH scale introduces uncertainty concerning the specificity of our data about graduate-level students. In future studies, it should be considered to include other instruments reporting on related aspects such as SF12/SF36 (mental health) [39], the Health Educations Impact Questionnaire (emotional distress) [40] or WHO-5 (well-being) [41].

The scales eHLA and eHLQ used to measure DL and eHL have only been applied in relatively few studies [24, 42] and not in relation to nursing students. It may be argued that the reliability and validity in our context has not yet been fully established.

## Conclusion

In general, the level of HL, DL and eHL is higher among graduate-level nursing students than in entry-level students. The average scores are at a satisfactory level which indicates that the current curriculum and study activities are appropriate but there is still room for improvement.

Age, sex, and social background, such as country of origin and parents’ educational and occupational background, influence students’ HL levels. Educators should be aware of this and how the diversity of the student group should be addressed by mixing the students in projects and group-based work to create a more inclusive environment.

## Supplementary information

**Additional file 1: Supplementary file 1.** Survey about Health Literacy and eHealth Literacy.

## Data Availability

The data generated and analyzed during the current study is not publicly available, due to containing information that could compromise research participants’ privacy and consent, but is available from the author DO if permission is obtained from the Danish Data Protection Agency.

## Abbreviations

AHLS: Adult Health Literacy Scale
DL: Digital Literacy
eHEALS: eHealth Literacy Scale
eHL: Electronic Health Literacy
eHLA: eHealth Literacy Assessment
eHLQ: eHealth Literacy Questionnaire.
HL: Health Literacy
HLQ: Health Literacy Questionnaire
IPR: Intellectual Property Rights
SRH: Self-rated health
WHO: World Health Organization
