# Supplementary material for: Health literacy, digital literacy and eHealth literacy in Danish nursing students at entry and graduate level: a cross sectional study
Source: BMC Nurs. 2020 Apr 10;19:22. doi: 10.1186/s12912-020-00418-w (PMC7149891; doi:10.1186/s12912-020-00418-w)
Supplement: Supplementary file 1 — Additional file 1: Supplementary file 1. Survey about Health Literacy and eHealth Literacy. [file 12912_2020_418_MOESM1_ESM.pdf]

1. Survey about Health Literacy and eHealth Literacy

Dear student

Thank you for participating in this survey. Your contribution is important.

Thank you for your input!

Kind regards

Project leader Kamilla Adellund Holt

Leader of Education, Lene Just

All the information, you give in the survey, will be anonymized and deleted upon finalization of the project. Data from the project will be handled in accordance with The Danish Data Protection Agency regulations by University College Copenhagen.

You declare, that you have received written information about the project and are informed about the objectives and methods.

You should know that it is voluntary to participate, and that you can always withdraw your declaration of consent without further notice.

By completing the questionnaire, you also consent to further processing of the data.

3. How old are you?

(Only enter one answer)

18	19	20	21	22	23	24	25	26	27	28	29
□	□	□	□	□	□	□	□	□	□	□	□

30+ ____________

4. What is your sex?

□ Male

□ Female

5. Country of birth

(Only enter one answer)

□ Denmark

Other ___________________________________

6. Do you speak Danish as a primary language at home?

(Only enter one answer)

□ Yes □ No

7. What is the highest level of education obtained by your parent?

(Choose the parent with the highest level of education)

(Only enter one answer)

□ Public school

□ General upper secondary education

□ Vocational training

□ Short-cycle higher education, below 3 yrs.

□ Medium-cycle higher education, 3-4 yrs.

□ Long-cycle higher education, above 5 yrs.

8. Is one of or both of your parents working/have worked in the social- and health sector?

(Only enter one answer)

Yes No

□ □

9. Have you been a patient at a hospital or received outpatient treatment?

(Only enter one answer)

Yes No

☐ ☐

10. Do you suffer from a chronic condition? (e.g. asthma, depression, eczema or diabetes)

(Only enter one answer)

No

☐

If yes, which?

____

____

____

____

11. Do you take prescribed medication daily? (Including birth control pills)

(Only enter one answer)

Yes No

☐ ☐

12. What is your level of education? (Choose your highest level of education)

(Only enter one answer)

☐ Public school

☐ General upper secondary education

☐ Vocational training

☐ Short-cycle higher education, below 3 yrs.

☐ Medium-cycle higher education, 3-4 yrs.

☐ Long-cycle higher education, above 5 yrs.

13. Overall, how would you rate your own health?

(Only enter one answer)

Excellent	Very good	Good	Less good	Poor
☐	☐	☐	☐	☐

14-48 eHLQ administered here. The questionnaire cannot be disclosed due to copyright and license restrictions.

Kayser L, Karnøe A, Furstrand D, Batterham R, Christensen KB, Elsworth G, Osborne RH. A Multidimensional Tool Based on the eHealth Literacy Framework: Development and Initial Validity Testing of the eHealth Literacy Questionnaire (EHLQ). Journal of Medical Internet Research 2018;20(2):e36. 10.2196/jmir.8371

49-94 HLQ administered here. The questionnaire cannot be disclosed due to copyright and license restrictions.

Osborne RH, Batterham RW, Elsworth GR, Hawkins M, Buchbinder R. The grounded psychometric development and initial validation of the Health Literacy Questionnaire (HLQ). BMC public health. 2013;13(1):658.

95-138 eHLA administered here. The questionnaire can be obtained in a Danish version or a preliminary version of the last author lk@sund.ku.dk
